# Identification of protein biomarkers in host cerebrospinal fluid for differential diagnosis of tuberculous meningitis and other meningitis

**DOI:** 10.3389/fneur.2022.886040

**Published:** 2022-08-08

**Authors:** Mailing Huang, Zeyu Ding, Wensheng Li, Weibi Chen, Yadong Du, Hongyan Jia, Qi Sun, Boping Du, Rongrong Wei, Aiying Xing, Qi Li, Naihui Chu, Liping Pan

**Affiliations:** ^1^Tuberculosis Department, Beijing Chest Hospital, Capital Medical University, Beijing, China; ^2^Beijing Tuberculosis and Thoracic Tumor Research Institute, Beijing, China; ^3^Neurology Department, Beijing Tiantan Hospital, Capital Medical University, Beijing, China; ^4^Department of Emergency Medicine, Beijing Chest Hospital, Capital Medical University, Beijing, China; ^5^Neurology Department, Xuanwu Hospital, Capital Medical University, Beijing, China; ^6^Beijing Key Laboratory for Drug Resistant Tuberculosis Research, Beijing Chest Hospital, Capital Medical University, Beijing, China

**Keywords:** tuberculous meningitis, infectious meningitis, cerebrospinal fluid, protein biomarker, diagnosis

## Abstract

**Background and purpose:**

The diagnosis of tuberculous meningitis (TBM) is difficult due to the lack of sensitive methods. Identification of TBM-specific biomarkers in the cerebrospinal fluid (CSF) may help diagnose and improve our understanding of TBM pathogenesis.

**Patients and methods:**

Of the 112 suspected patients with TBM prospectively enrolled in the study, 32 patients with inconclusive diagnosis, non-infectious meningitis, and long-term treatment with hormones and immunosuppressants were excluded. The expression of 8 proteins in the CSF was analyzed using ELISA in 22 patients with definite TBM, 18 patients with probable TBM, and 40 patients with non-TBM.

**Results:**

Significant differences in the expression of 7 proteins were detected between the TBM and non-TBM groups (*P* < 0.01). Unsupervised hierarchical clustering (UHC) analysis revealed a disease-specific profile consisting of 7 differentially expressed proteins for TBM diagnosis, with an accuracy of 82.5% (66/80). Logistic regression with forward stepwise analysis indicated that a combination of 3 biomarkers (APOE_APOAI_S100A8) showed a better ability to discriminate TBM from patients with non-TBM [area under the curve (AUC) = 0.916 (95%CI: 0.857–0.976)], with a sensitivity of 95.0% (95%CI: 83.1–99.4%) and a specificity of 77.5% (95%CI: 61.5–89.2%).

**Conclusion:**

Our results confirmed the potential ability of CSF proteins to distinguish TBM from patients with non-TBM and provided a useful panel for the diagnosis of TBM.

## Introduction

Tuberculous meningitis (TBM) is a common form of meningitis caused by infection of *Mycobacterium tuberculosis* (*M.TB*) and is the most severe form of tuberculosis (TB). Although the proportion of TBM may only be 1–5% of all TB cases (1–3), its mortality is unacceptably high (range 10–36.5%) (2, 3), especially in developing countries. Furthermore, survivors often experience neurological sequelae, which seriously influence their quality of life (4, 5). In most cases, TBM develops rapidly to a severe form within a few months after *M.TB* infection (6). Early diagnosis and timely adequate treatment are the most significant factors in reducing morbidity, mortality, and healthcare costs (5, 7).

Since the clinical manifestations of TBM are similar to other infectious meningitis, including viral meningitis (VM), bacterial meningitis (BM), and cryptococcal meningitis (CM), it is difficult to distinguish TBM from other infectious forms of meningitis. Furthermore, laboratory diagnostic methods, including smear microscopy of cerebrospinal fluid (CSF), CSF *M.TB* culture, and other commercial nucleic acid amplification technique (NAAT) tests targeting *M.TB*, do not present sufficient sensitivity for the identification of TBM. All these reasons make the diagnosis of TBM extremely difficult (8–10), leading to a high rate of misdiagnosis of TBM with other infectious meningitis and leading to fatal outcomes in clinical practice. Therefore, a novel technique is urgently needed for the diagnosis of TBM.

The World Health Organization (WHO) announced that non-sputum diagnostic biomarkers for TB should be developed (11), and subsequently, several TB biomarkers have been reported using high-throughput proteomics or transcriptomics analysis. The disease process of TBM differed widely between the periphery and the central nervous system (12). The CSF is constantly being exchanged with the interstitial fluid of the brain, which allows changes in the protein profile following *M.TB* infection to be closely reflected in the CSF; and renders the CSF a promising source of biomarkers for TBM (13).

Previous studies have identified potential protein biomarkers present in the CSF for the diagnosis of TBM using mass spectrometry (MS)-based high-throughput proteomics technologies (14–17). However, the results of these studies have not been translated into clinical practice so far, due to their poor generalizability. To date, four different studies have identified three sets of differentially expressed proteins in the CSF to distinguish TBM from other meningitis diseases or healthy controls, but there is limited overlap among the four studies. The high heterogeneity among different studies may be due to the different enrollment criteria for the TBM and control groups, as well as the insufficient sample sizes applied in the proteomic stage. Therefore, further validation of proteomic results is critical for the exploration of biomarkers. However, limited validation information is available in these studies.

We hypothesized that at least some of these biomarkers, especially those repeatedly identified in different studies, will have greater potential in the diagnosis of TBM. Therefore, we aimed to evaluate the usefulness of previously identified potential CSF biomarkers in a new cohort of immunocompetent adults with suspected TBM and to build a diagnostic panel for the diagnosis of TBM.

## Materials and methods

### Study population and ethical approval

Patients with suspected TBM who had a headache or altered mental status with clinical syndrome or signs suggestive of TBM were prospectively and consecutively enrolled between February 2017 and September 2019, at the Beijing Chest Hospital, Beijing Tiantan Hospital, and Beijing Xuanwu Hospital. Routine clinical investigations, including brain imaging, chest radiography, abdominal ultrasonography, sputum and/or CSF Xpert MTB/RIF, smear, *M.TB* culture, or other PCR tests, were performed as clinically indicated. Finally, the clinical history, physical examination, and detailed neurological assessment findings were prospectively recorded in agreement with the data that were considered important by Marais and colleagues (18). Patients with anti-TB treatment > 14 days or anti-infection treatment > 3 days were not enrolled.

The study was performed in accordance with the guidelines of the Declaration of Helsinki and its later amendments or comparable ethical standards and was approved by the Ethics Committee of the Beijing Chest Hospital, Capital Medical University (No. BJXK-2017-37-02). Written informed consent was obtained from each participant.

### Categorization of patients

The final diagnosis was done based on clinical manifestation, routine biochemical examinations, and radiological, histopathological, and microbiological information. Patients were defined as (1) definite TBM: patients had symptoms or signs of TBM and presented positive acid-fast bacilli, positive *M.TB* culture, positive Xpert MTB/RIF test, or positive PCR tests for *M.TB* in the CSF; (2) probable TBM: patients had a diagnostic score of ≥ 12 when cerebral imaging was available and a diagnostic score of ≥ 10 when cerebral imaging was unavailable; and (3) possible TBM: with cerebral imaging available, a diagnostic score of 6–11 was required, and with cerebral imaging unavailable, a score of 6–9 was required. The diagnostic score was based on the uniform clinical case definition recommended by previous studies (18, 19). Briefly, the diagnostic scoring system included the presence of symptoms or signs indicative of meningitis plus four additional levels of information, namely, clinical criteria, CSF criteria, cerebral imaging criteria, and evidence of TB elsewhere. (4) Non-TBM: an alternative cause for meningitis was identified by microbiological, histopathological, and serological examinations and the response to appropriate nontuberculous therapy, including other infectious meningitis, such as virus meningitis, CM, and BM (20, 21).

### Selection of target CSF proteins

We searched PubMed for the terms “tuberculous meningitis,” “cerebrospinal fluid,” “proteomic,” “proteome,” and “Mass spectrometry” and found only 4 publications up to 2019 (14–17). The biomarkers of the CSF protein with a fold change of > 2 in each study, which were repeatedly identified in at least two studies and presented consistent regulation patterns in TBM compared to controls, were selected for validation. A total of 8 CSF proteins were selected for our study, including Alpha-1-Antichymotrypsin (ACT), Anti-thrombin III, Haptoglobin, Apolipoprotein AI (APOAI), Apolipoprotein B (APOB), Apolipoprotein E (APOE), S100 Calcium-Binding Protein A8 (S100A8), and Transthyretin.

### Specimen collection

Cerebrospinal fluid samples (~1–3 ml) were collected from patients with suspected TBM during a routine diagnostic evaluation at admission. All samples were centrifuged at 2,000 × *g* for 10 min at 4°C, aliquoted in sterile polypropylene microtubes, and stored at −80°C until use.

### ELISA analysis

The Human ACT ELISA Kit (ab157706; Abcam), Human Anti-Thrombin III ELISA Kit (ab108801; Abcam), Human Haptoglobin ELISA Kit (ab108858; Abcam), Human APOAI ELISA Kit (ab189576; Abcam), Human APOB ELISA Kit (ab108807; Abcam), Human APOE ELISA Kit (ab108813; Abcam), Human S100A8 ELISA Kit (ab267628; Abcam), and Human Transthyretin ELISA Kit (ab108895; Abcam) were used according to the manufacturers' instructions to measure protein concentrations in the CSF. Throughout the study, clinicians were blinded to ELISA results and laboratory technicians were blinded to diagnosis. Thus, laboratory interpretation and diagnosis were independent of test results.

### Data analysis

Data analysis by Mu and colleagues showed that the APOB performance of CSF gave a sensitivity of 89.3% and a specificity of 92% in the diagnosis of TBM, respectively (15). APOB was also included in our study; therefore, the abovementioned sensitivity and specificity were defined as assuming parameters to calculate the sample size, with an allowable error of 10%, a significance level of 5%, and the sample size of TBM and non-TBM was matched by a ratio of 1:1. Furthermore, considering possible missing data, the sample size was expanded by 10%. Finally, it was calculated that 40 samples in each group were optimal.

Continuous variables were tested using Student's *t* test or Mann–Whitney *U* test, while categorized variables were analyzed using Fisher's exact test or Pearson's chi-square test. The accordance of the expression levels among the proteins was analyzed by the Pearson correlation test. Logistic regression with forward stepwise analysis was used to establish the diagnostic panel. The receiver operating characteristic curves (ROCs) were constructed to obtain the area under the curve (AUC) and evaluate the diagnostic values of the single biomarker and the panel. *P* < 0.05 was considered statistically significant. All these data analyses were performed using SPSS version 21.0 (SPSS Inc., Chicago, IL, USA) and GraphPad Prism version 5.0 (Graph Pad Software Inc., San Diego, CA, USA). Unsupervised hierarchical clustering (UHC) and principal component analysis (PCA) were performed using the MetaboAnalyst version 4.0 software (https://www.metaboanalyst.ca/). The biological signaling pathway analysis was performed with the Kyoto Encyclopedia of Genes and Genomes (KEGG) database (http://www.genome.jp/kegg/pathway.html/).

## Results

### Demographic and clinical information of the study population

**Figure 1:**
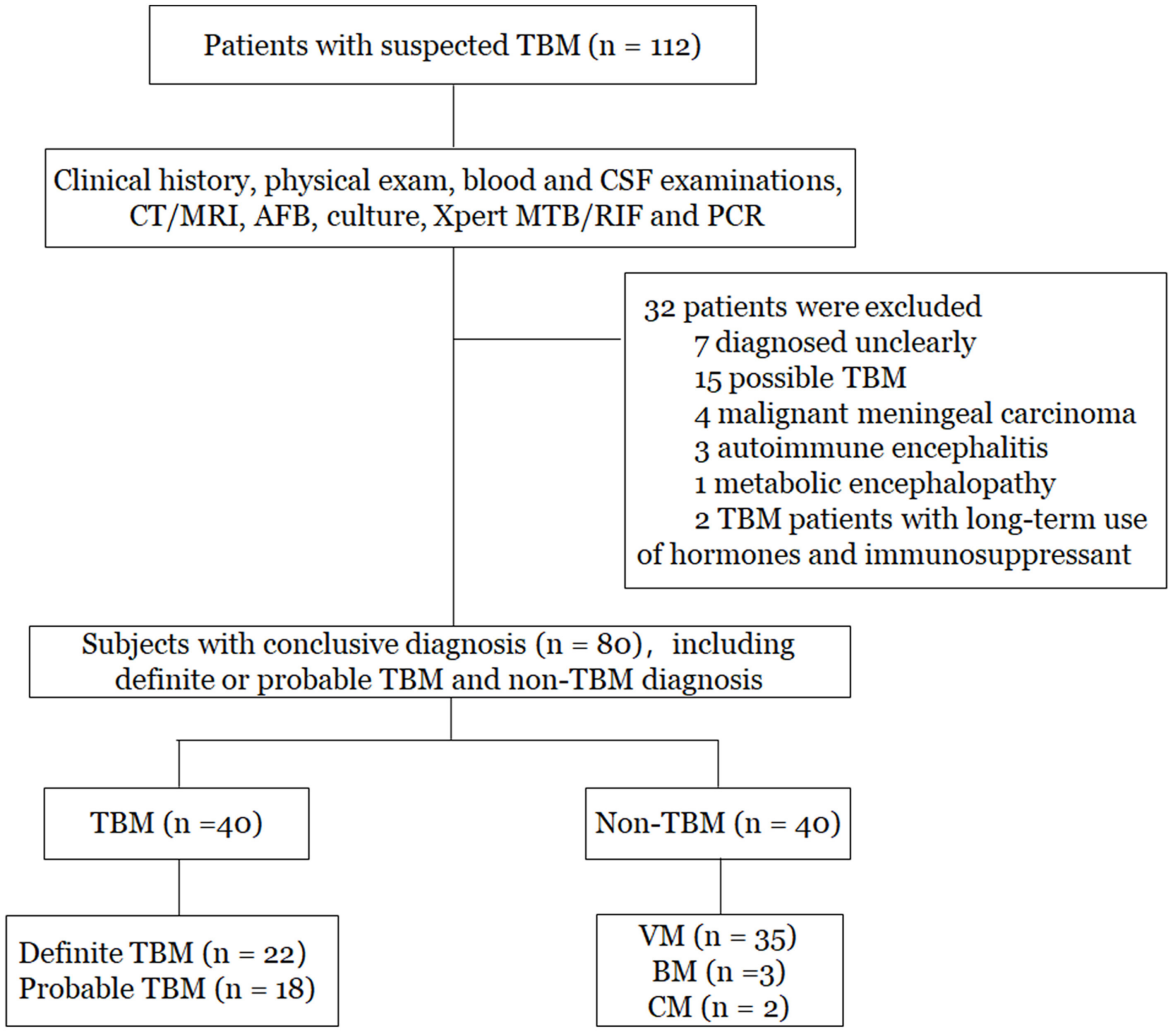
Flowchart of the study participants. TBM, tuberculous meningitis; VM, viral meningitis; BM, bacterial meningitis; CM, cryptococcal meningitis; CT, computed tomography; MRI, magnetic resonance imaging; PCR, polymerase chain reaction.

As shown in Figure 1, a total of 112 patients with suspected TBM were initially enrolled. Based on the categorization of the patients, 80 patients who had conclusive diagnostic information on infectious meningitis were included in the study. The remaining 32 patients were excluded due to unclear diagnosis (*n* = 7), possible TBM (*n* = 15), malignant meningeal carcinoma (*n* = 4), autoimmune encephalitis (*n* = 3), metabolic encephalopathy (*n* = 1), and long-term treatment with hormones and immunosuppressants (*n* = 2). Finally, among the 80 patients included in the study, there were 22 patients with definite TBM, 18 patients with probable TBM, and 40 patients with non-TBM. Among the non-TBM group, there were 35 patients with VM, 3 patients with BM, and 2 patients with CM.

The demographic and clinical information of the 80 patients in the study were described in Table 1. No significant differences in age or gender were detected between the TBM and non-TBM groups (*P* > 0.05). There were no significant differences in the underlying diseases of the patients between the TBM and non-TBM groups (*P* > 0.05). More patients with TBM presented the symptom of fever (*P* = 0.034) and vomiting (*P* = 0.021), while more patients with non-TBM presented the symptom of convulsions (*P* = 0.045). No patient had HIV infection in the TBM and non-TBM groups. Furthermore, patients with TBM showed significantly lower glucose and chloride concentrations, as well as a lower proportion of monocytes in the CSF than those of patients with non-TBM (*P* < 0.001), while significantly higher protein concentrations were detected in the CSF in patients with TBM than that in patients with non-TBM.

**Table 1:** Demographic and baseline clinical characteristics of participants*.

**Characteristics**	**TBM^#^ group**	**Non-TBM^&^ Group**	***P*-Value**
	**(*n =* 40)**	**(*n =* 40)**	
Age, yrs	29 (22–54)	41 (26–54)	0.137
Gender (female/male)	16/24	14/26	0.644
HIV infection	0 (0)	0 (0)	1.000
**Underlying diseases**			
Diabetes mellitus	3 (7.5)	1 (2.5)	0.615
Hypertension	4 (10.0)	6 (15.0)	0.499
None	30 (75.0)	29 (70.0)	0.799
**Presenting symptoms**			
Fever	37 (92.5)	30(75.0)	0.034
Headache	29 (72.5)	22 (55.0)	0.104
Vomiting	20 (50.0)	11 (27.5)	0.001
Convulsion	4 (10.0)	11(27.5)	0.045
Consciousness disorder	18 (45.0)	14 (35.0)	0.361
**Cerebrospinal fluid examination**			
Cerebrospinal fluid clear appearance	37 (92.5)	37 (92.5)	1.000
Total white blood cells count, cell ×10^3^/mL	174.5 (98.8–417.8)	26.5 (6.2–160.8)	<0.001
Monocytes proportion, %	44.3 (26.2–66.7)	97.9 (89.0–100.0)	<0.001
Protein level, mmol/L	141.1 (97.5–217.2)	42.0 (28.6–66.2)	<0.001
Glucose, mg/dL	1.8 (0.9–2.4)	3.3 (2.8–4.1)	<0.001
Chloride concentration, mmol/L	112.4 (105.0–119.8)	125.0 (121.0–128.0)	<0.001

*Data are median (interquartile range, IQR) or n (%).

^#^TBM includes definite and probable cases.

^&^Non-TBM includes viral meningitis, bacterial meningitis and cryptococcal meningitis.

Detailed information on patients with TBM is described in Table 2. Overall, 65% of patients with TBM presented symptoms for more than 2 weeks. Only 25% of patients did not receive anti-TB or hormone treatment, while the remaining 75% (*n* = 30) of patients received anti-TB treatment (e.g., isoniazid, rifampicin, pyrazinamide, and ethambutol) and prednisone for 1–14 days. A total of 17 patients were defined in stage I, 5 patients were defined in stage II, and 18 patients were defined in stage III by the British Medical Research Council (MRC) clinical stage of TBM (22).

**Table 2:** Characteristics of patients with TBM^#^.

**Characteristics**	**No. (%)**
Total No.	40 (100.0)
**History**	
Tuberculosis contact in history	2 (5.0)
Symptom duration > 14 d	26 (65.0)
Symptom duration 1–14 d	14 (35.0)
With anti-TB treatment duration 1–14 d	30 (75.0)
Without anti-TB treatment	10 (25.0)
**TBM category**	
Definite TBM	22 (55.0)
Probable TBM	18 (45.0)
TBM stage*	
I	17 (42.5)
II	5 (12.5)
III	18 (45.0)
**Cerebral imaging**	
Hydrocephalus	5 (12.5)
Basal meningeal enhancement	9 (22.5)
Tuberculoma	22(55.0)
Infarct	9 (22.5)

^#^Data are n (%).

*TBM stage was defined by British Medical Research Council clinical stage of TBM (22).

### The expression level of proteins in the CSF between TBM and non-TBM

**Figure 2:**
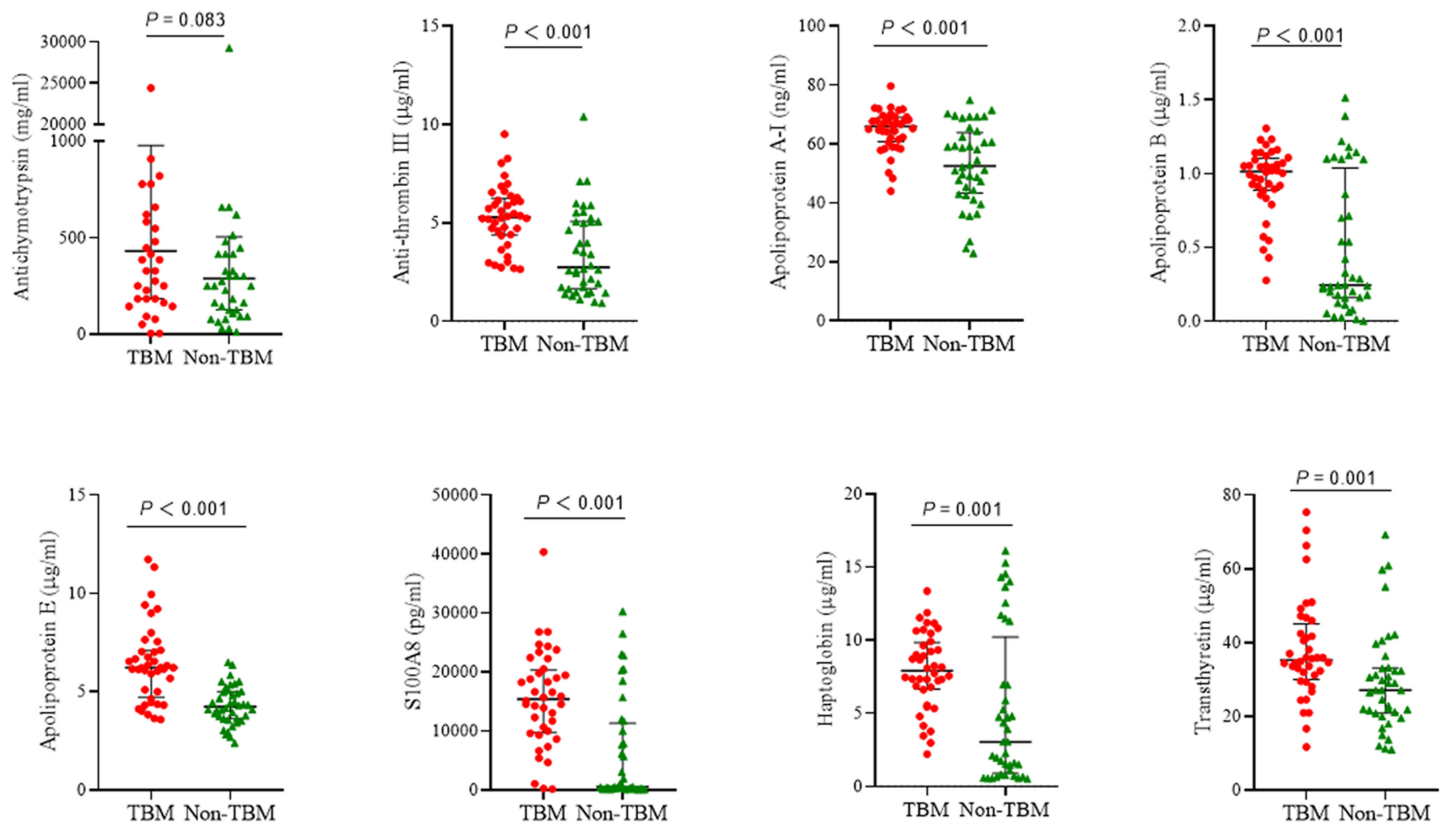
The expression of 8 proteins in the cerebrospinal fluid between patients with TBM and non-TBM. TBM, tuberculous meningitis.

**Figure 3:**
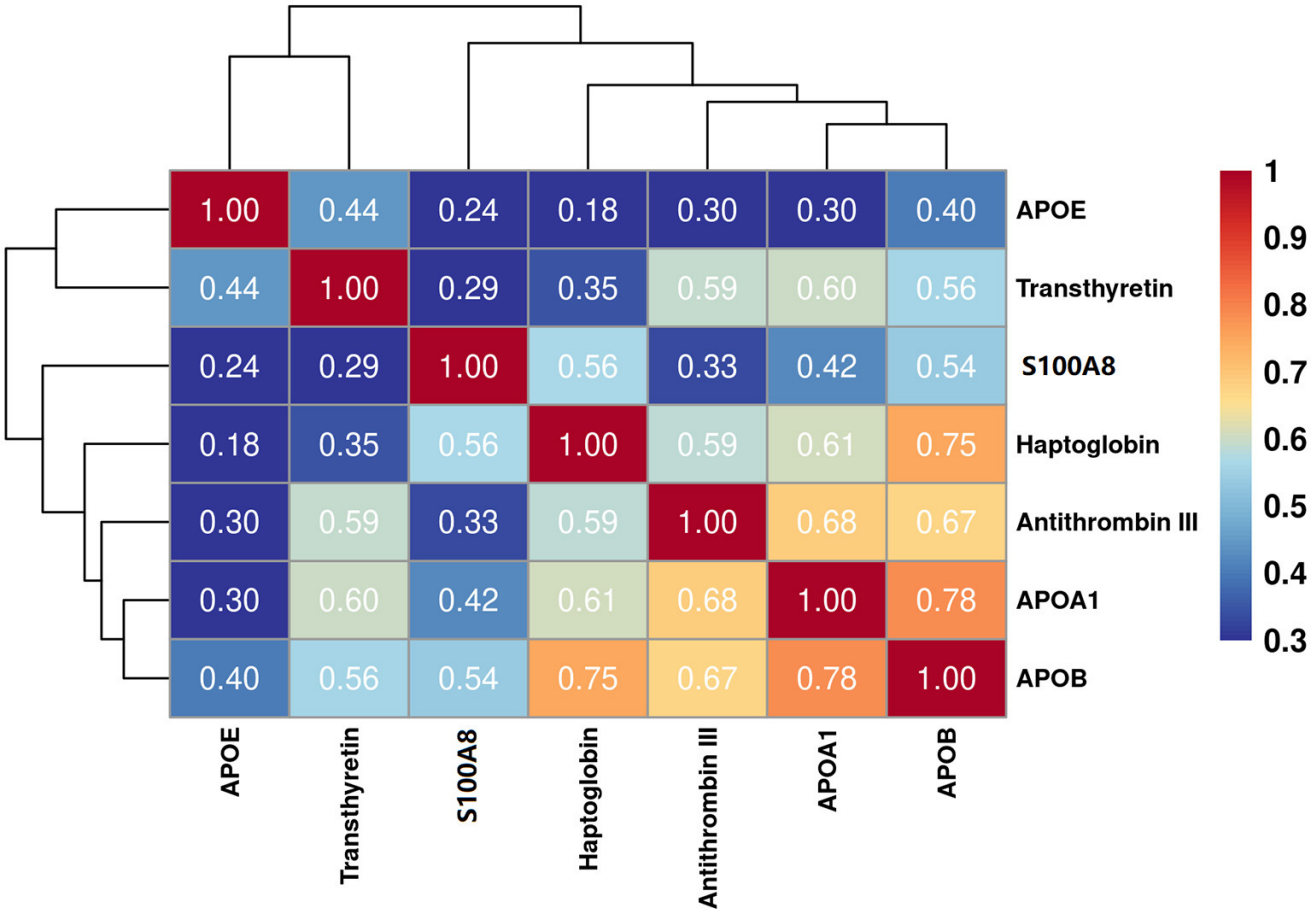
Partial correlation analysis of the 7 differentially expressed proteins. The correlation values between any two proteins were presented in the figure.

The eight target proteins were analyzed using ELISA tests. As shown in Figure 2 and Supplementary Table 1, the concentration of Anti-thrombin III, APOAI, APOB, APOE, S100A8, Haptoglobin, and Transthyretin was significantly higher in the TBM group than those in the non-TBM group (*P* < 0.01). No significant differences were detected in the expression level of ACT between TBM and non-TBM groups (*P* = 0.083). The correlation analysis showed that APOB had a significant correlation with APOAI and Haptoglobin, respectively, Anti-thrombin III had a moderate correlation with APOB and APOAI, respectively, and a weak correlation was detected among other proteins (Figure 3).

The subgroup analyses of the TBM group are presented in Supplementary Figures 1–3. No significant differences were detected in the expression level of eight biomarkers between definite and probable TBM patients. Furthermore, there were also no significant differences in biomarker expression between patients with different durations of symptoms or between patients with different stages of TBM.

### Bioinformatics analysis

**Figure 4:**
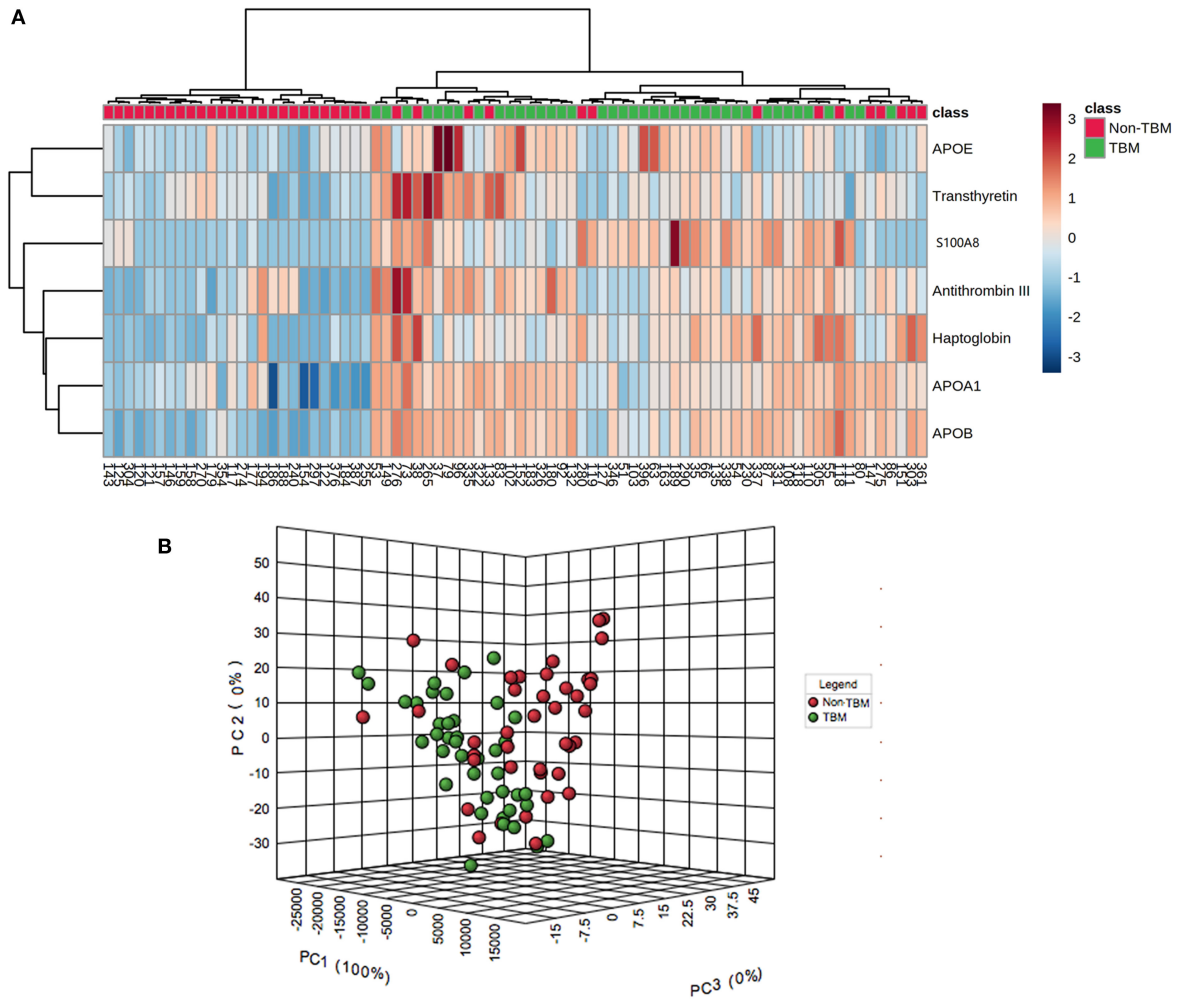
Unsupervised hierarchical clustering and principal component analyses of differentially expressed proteins in the CSF between patients with TBM and non-TBM. **(A)** Two-dimensional unsupervised hierarchical clustering of proteins in CSF between patients with TBM and non-TBM. The normalized values for each protein are depicted in accordance with the color scale, where red and blue represent upregulation and downregulation, respectively. **(B)** Three-dimensional representation of principal component analysis (PCA) of patients with and without TBM. Each dot represents 1 participant based on the values of all proteins studied. Green and red represent patients with TBM and non-TBM, respectively. TBM, tuberculous meningitis; CSF, cerebrospinal fluid.

To identify whether the 7 differentially expressed proteins in the CSF could be indicative of a TBM-specific profile, UHC and PCA analyses were performed based on the expression of the 7 proteins. The UHC analysis showed that a significant clustering of patients with TBM was evidenced, while 14 patients with non-TBM were incorrectly clustered into the TBM group (Figure 4A), including 10 patients with VM, 3 patients with BM, and 1 patient with CM. The accuracy of this TBM-specific profile was 82.5% (66/80). The results of the UHC analysis were also confirmed by the PCA analysis, with some non-TBM patients presented closer distance with TBM patients (Figure 4B). To better understand the biological relevance of the 7 differentially expressed proteins in CSF with TBM, the KEGG pathway analysis was performed (Supplementary Figure 4). These proteins were matched in pathways including cholesterol metabolism (APOAI, APOB, and APOE), vitamin digestion and absorption (APOAI and APOB), fat digestion and absorption (APOAI and APOB), peroxisome proliferator-activated receptor signaling (APOAI), interleukin-17 signaling (S100A8), complement and coagulation cascades (Anti-Thrombin III), thyroid hormone synthesis (Transthyretin), African trypanosomiasis (APOAI), and Alzheimer's disease (APOE). The representative proteins expressed in these pathways had higher expression in the TBM group.

### Diagnostic values of protein biomarkers to distinguish TBM from non-TBM

**Figure 5:**
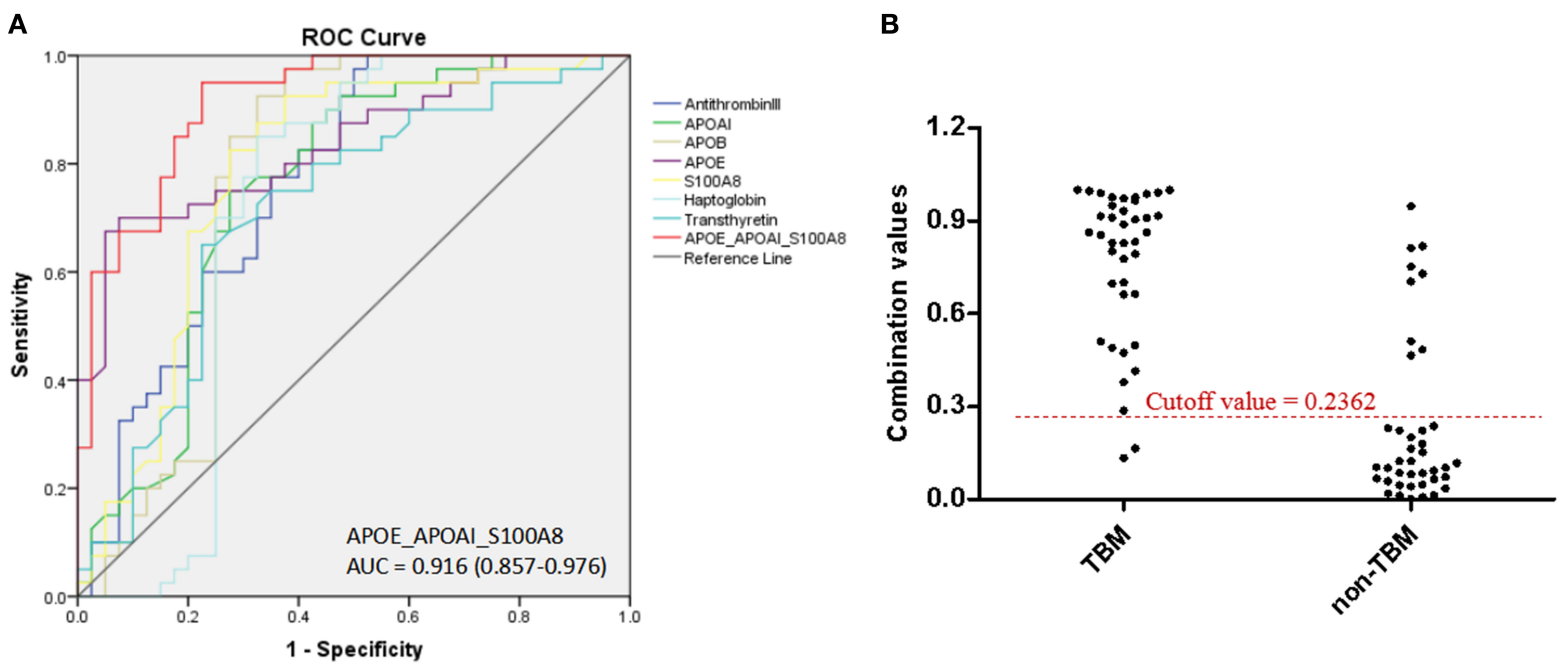
The diagnostic value of CSF proteins and the panel (APOAI_APOE_S100A8) in distinguishing TBM from patients with non-TBM. **(A)** The receiver operating characteristic curve (ROC) depicts the sensitivity and specificity of the proteins and the panel in CSF in distinguishing TBM from patients with non-TBM. **(B)** Scatter plot showing the ability of the diagnostic panel (APOAI_APOE_S100A8) in distinguishing TBM from patients with non-TBM. TBM, tuberculous meningitis; CSF, cerebrospinal fluid.

Based on the UHC and PCA analyses, a specific CSF protein profile consisting of 7 differentially expressed proteins in CSF for the diagnosis of TBM was detected. However, whether the 7 differentially expressed proteins were essential biomarkers for TBM diagnosis should be further analyzed. The ROC analysis was performed to evaluate the discriminative capacity of these 7 biomarkers (Table 3, Figure 5). The AUC of these 7 biomarkers ranged from 0.712 to 0.838 for discriminating TBM from non-TBM. APOE was the best biomarker to distinguish TBM from non-TBM (AUC = 0.838). Logistic regression with forward stepwise analysis indicated that a combination of 3 biomarkers (APOAI_APOE_S100A8) could show a better discrimination ability for discriminating TBM from non-TBM [AUC = 0.916 (95%CI = 0.857–0.976)], with a sensitivity of 95.0% (95%CI = 83.1–99.4%) and a specificity of 77.5% (95%CI = 61.5–89.2%).

**Table 3:** ROC analyses of the CSF protein profile for discriminating TBM from non-TBM diseases.

	**Cut-off value**	**TBM** ***n* = 40**	**Non-TBM** ***n* = 40**	**AUC (95%CI)**	**Sensitivity% (95%CI)**	**Specificity% (95%CI)**	**NPV (95%CI)**	**PPV (95%CI)**	**LR+ (95%CI)**	**LR- (95%CI)**
Anti-thrombin III	> 2.61	40/40	19/40	0.766 (0.658–0.854)	100.0 (91.2–100.0)	47.5 (31.5–63.9)	/†	65.6 (58.7–71.9)	1.9 (1.4–2.6)	/†
APOAI	> 60.67	30/40	29/40	0.753 (0.644–0.842)	75.0 (58.8–87.3)	72.5 (56.1–85.4)	74.4 (62.1–83.7)	73.2 (61.5–82.3)	2.7 (1.6–4.7)	0.3 (0.2–0.6)
APOB	> 0.42	39/40	39/40	0.769 (0.661–0.856)	97.5 (86.8–99.9)	62.5 (45.8–77.3)	90.0 (74.8–96.5)	74.0 (64.4–81.8)	2.8 (1.8–4.5)	0.1 (0.04–0.3)
APOE	> 5.51	28/40	28/40	0.838 (0.739–0.911)	70.0 (53.5–83.4)	92.5 (79.6–98.4)	75.5 (65.5–83.3)	90.3 (75.5–96.6)	9.3 (3.1–28.2)	0.3 (0.2–0.5)
S100A8	> 3058.09	37/40	37/40	0.783 (0.677–0.867)	92.5 (79.6–98.4)	62.5 (45.8–77.3)	89.3 (73.2–96.2)	71.2 (62.1–78.8)	2.5 (1.6–3.7)	0.1 (0.04–0.3)
Haptoglobin	> 5.27	34/40	27/40	0.712 (0.600–0.808)	85.0 (70.2–94.3)	67.5 (50.9–81.4)	72.3 (62.2–80.6)	81.8 (67.6–90.7)	2.6 (1.6–4.2)	0.2 (0.1–0.5)
Transthyretin	> 33.17	26/40	31/40	0.713 (0.601–0.809)	65.0 (48.3–79.4)	77.5 (61.5–89.2)	74.3 (60.9–84.3)	68.9 (58.4–77.7)	2.9 (1.6–5.4)	0.5 (0.3–0.7)
APOAI_APOE_ S100A8*	> 0.236	38/40	31/40	0.916 (0.857–0.976)	95.0 (83.1–99.4)	77.5 (61.5–89.2)	80.9 (70.3–88.3)	93.9 (79.9–98.4)	4.2 (2.4–7.5)	0.06 (0.02–0.3)

*The combination was consisted of APOAI, APOE and S100A8 in the CSF.

†The NPV and LR+ value were unavailable when the diagnostic sensitivity was 100%.

TBM, tuberculous meningitis; ROC, receiver operating characteristic curve; AUC, the area under the ROC curve; NPV, negative predictive value; PPV, positive predictive value; LR+, likelihood ratio for positive test; LR–, likelihood ratio for negative value.

## Discussion

The rapid development of proteomics and transcriptomics has paved the way for the identification of disease biomarkers. Many differentially expressed proteins have been identified as TBM-specific proteins by MS technologies in blood or CSF. However, the verification of biomarkers in independent sets is of great importance before the clinical application of these biomarkers, to improve the reproducibility and accuracy (23). In our study, we searched for studies based on CSF proteomics analysis for TBM until 2019 and found that more than 100 differentially expressed proteins were detected in previous proteomics studies. However, most differentially expressed proteins were not validated and only a few overlapped proteins were identified across the different studies, presenting confusion in the clinical use of these biomarkers. Therefore, differentially expressed proteins in at least two studies were selected and further validated in a new cohort with suspected TBM, to confirm the usefulness of these biomarkers, and constructed a useful diagnostic panel for the diagnosis of TBM.

Among the 8 repeated expressed proteins identified in a previous proteomics study in CSF, 7 were differentially expressed between the TBM and non-TBM groups in our study, including Anti-Thrombin III, Haptoglobin, APOAI, APOB, APOE, S100A8, and Transthyretin. The expression of these 7 proteins was significantly higher in the TBM group than those of the non-TBM group, which was consistent with previous proteomics studies (14–17). However, no significant differences were detected in the expression of ACT in our study. As we know, ACT is an acute-phase protein involved in various inflammatory conditions. Previous studies have also suggested that the expression of ACT in plasma or serum was generally higher in patients with TB compared to healthy controls (24, 25). Nevertheless, patients with VM, BM, or CM (not healthy controls) were included in the non-TBM group in our study. They were all infectious meningitis; infection with virus, bacteria, or fungus inevitably activates the host inflammation response. Therefore, it is reasonable that no significant differences in the expression level of ACT between the TBM were detected in the non-TBM group in our study.

Using UHC and PCA analyses, we identified a specific TBM profile consisting of 7 proteins in the CSF for patients with TBM compared to other types of infectious meningitis. Based on this profile, all patients with TBM were correctly distinguished, but all the 3 patients with BM were incorrectly grouped with the patients with TBM. The result suggested that the pathological mechanisms and processes of these two diseases shared more similarities, compared to VM and CM. Essentially, *M.TB* also belonged to the category of bacterial infections, despite the differences in treatment regimens. Furthermore, a previous study has also suggested that the transcriptomics profiles of some probable TBM patients were more similar to those of BM (12).

We evaluated the diagnostic capacity of the 7 CSF proteins and found that all proteins had AUC values greater than 0.7, which indicated the potential to distinguish TBM from non-TBM. Immunological response of patients with TBM or non-TBM is comprehensive and complex. It is unrealistic to rely on a single protein to accurately distinguish TBM from non-TBM. Combining multiple differentially expressed proteins can achieve better diagnostic performance. However, a specific profile consisting of too many biomarkers will be limited in its clinical application. In this field, a minimal set of proteins with higher diagnostic accuracy is urgently needed. Therefore, we performed logistic regression analysis and identified a panel consisting of only three proteins (APOAI_APOE_S100A8), which could enhance the diagnostic accuracy to 86.3% for distinguishing TBM from non-TBM, with a sensitivity of 95.0% and a specificity of 77.5%.

The distinctive lipid composition in the cell envelope of *M.TB* makes it unique from other bacteria and facilitates intracellular survival. Consequently, host proteins involved in lipid metabolism could be regulated to adapt or resist invasion by *M.TB* (26, 27). APOAI is the main protein component of high-density lipoprotein. Serum APOAI has also been reported to significantly increase in pediatric patients with TBM compared to patients with other infectious meningitis, which was consistent with the findings of our study (28). APOE is mainly released by astrocytes, oligodendrocytes, and neurons in the human brain and is involved in maintaining cholesterol and phospholipid homeostasis (29). A previous study has demonstrated that the deficiency of the *APOE* gene could highly increase the susceptibility to *M.TB* infection, indicating that APOE may be involved in the occurrence and development of TB (30). Except for these two apolipoproteins, APOB also significantly presented a higher expression level in the TBM CSF than that in the non-TBM CSF, although it was not included in the final diagnostic panel. A significantly higher expression of APOAI, APOB, and APOE in patients with TBM than those with other infectious meningitis indicated that the occurrence and progression of TBM are related to abnormal lipid metabolism.

S100A8 belongs to the S100 protein family and is primarily expressed by neutrophils and monocytes. It is a feature of chronic inflammatory diseases, such as autoimmune diseases and TB. Previous studies have confirmed the involvement of S100A8 in the occurrence and development of TB, by recruiting neutrophils and mediating inflammation in TB (31, 32). S100A8 is closely associated with central nervous system inflammation, especially with immune reconstitution inflammatory syndrome (IRIS) in TBM (33). Biomarker studies have also determined that S100A8 is differentially upregulated in patients with tuberculous pleurisy compared to patients with cancer (34). In our study, it was confirmed that patients with TBM had higher levels of S100A8 in the CSF than patients with non-TBM, and finally, S100A8 was included in the diagnostic panel. Thus, S100A8 is related to the pathological and biological progression of TBM.

Identification of TBM-specific proteins in CSF using proteomics technologies is an effective and useful approach for the diagnosis of TBM, but it is worth noting that validation of these potential biomarkers in independent sets is of great importance. To the best of our knowledge, only four proteomics studies have been performed to date that identified potential protein biomarkers in the CSF for the diagnosis of TBM, and most studies have not been verified in large independent sets. The protein biomarkers overlapped between these studies may have a significant potential for the diagnosis of TBM, so we selected them for further validation and confirmed the feasibility of these biomarkers for the diagnosis of TBM. However, there are still some limitations in our study. Due to the limited CSF proteomics studies until now, only 8 overlapped proteins were selected for validation. We could not rule out the fact that other proteins identified in these proteomics studies may also be potential biomarkers. Furthermore, because of the low incidence of infectious meningitis, the sample size in our study was not large enough. In addition, due to the use of vaccines and antibiotics, the incidence of BM decreased, especially in adults. CM often occurred in patients with HIV infection or another immunosuppressive status, and the incidence of CM in China was also not high (35, 36). Thus, the differentiation between TBM and VM has become the most important and urgent issue in clinical practice. Therefore, the majority of patients in the non-TBM group were patients with VM. However, further validations including a large number of patients with different types of infectious meningitis and other non-infectious meningitic diseases should be performed in future, in order to confirm the effective application of these biomarkers in clinical practice.

In conclusion, our study validated 8 overlapped proteins identified in previous proteomics studies and confirmed that the expression level of 7 proteins was significantly different between the TBM and non-TBM groups. Furthermore, a diagnostic panel consisting of APOAI_APOE_S100A8 was constructed and presented a relatively good capacity to distinguish patients with TBM from patients with non-TBM.

## Data availability statement

The original contributions presented in the study are included in the article/Supplementary material, further inquiries can be directed to the corresponding author.

## Ethics statement

The studies involving human participants were reviewed and approved by the Ethics Committee of Beijing Chest Hospital, Capital Medical University. The patients/participants provided their written informed consent to participate in this study.

## Author contributions

MH, LP, ZD, NC, and QL conceived the study. MH, ZD, and LP analyzed the results and wrote the manuscript. MH, HJ, QS, BD, RW, and AX performed the experiment. MH, ZD, WL, WC, and YD participated in the recruitment and collection of medical records. All authors contributed to the article and approved the submitted version.

## Funding

This study was financially supported by grants from the China Tuberculosis Clinical Trial Consortium (CTCTC) (2017KYJJ004), the Beijing Natural Science Foundation (7192038), the National Natural Science Foundation (81902024), and the Tongzhou Yunhe Project (YH201807 and YH201906).

## Conflict of interest

The authors declare that the research was conducted in the absence of any commercial or financial relationships that could be construed as a potential conflict of interest.

## Publisher's note

All claims expressed in this article are solely those of the authors and do not necessarily represent those of their affiliated organizations, or those of the publisher, the editors and the reviewers. Any product that may be evaluated in this article, or claim that may be made by its manufacturer, is not guaranteed or endorsed by the publisher.

## Supplementary material

Supplementary Table 1 The expression of 8 proteins in CSF between patients with TBM and non-TBM.

Supplementary Figure 1 The expression of 8 proteins in CSF between definite and probable TBM patients.

Supplementary Figure 2 The expression of 8 proteins in patients with TBM with different symptom durations.

Supplementary Figure 3 The expression of 8 proteins in patients with TBM with different disease stages.

Supplementary Figure 4 KEGG analysis of 7 differentially expressed proteins.
