# Supplementary material for: Identification of protein biomarkers in host cerebrospinal fluid for differential diagnosis of tuberculous meningitis and other meningitis
Source: Front Neurol. 2022 Aug 8;13:886040. doi: 10.3389/fneur.2022.886040 (PMC9393334; doi:10.3389/fneur.2022.886040)
Supplement: Supplementary Table 1 — The expression of 8 proteins in CSF between patients with TBM and non-TBM. [file Table_1.DOCX]

**Supplementary Table 1. The expressed level of the 8 proteins in CSF between TBM and Non-TBM patients between TBM and Non-TBM patients**

| Proteins | TBM (n = 40) | Non-TBM (n = 40) | *P*-values |
| --- | --- | --- | --- |
| Antichymotrypsin (mg/mL) | 431.4(183.4-977.4) | 288.6(125.7-504.6) | 0.083 |
| Anti-thrombin III (μg/mL) | 5.3(4.4-6.2) | 2.7(1.7-5.1) | **＜0.001** |
| Apolipoprotein A-I (ng/mL) | 66.0(60.8-69.1) | 52.5(43.3-63.9) | **＜0.001** |
| Apolipoprotein B (μg/mL) | 1.0(0.9-1.1) | 0.2(0.2-1.0) | **＜0.001** |
| Apolipoprotein E (μg/mL) | 6.2(4.7-7.1) | 4.2(3.6-5.0) | **＜0.001** |
| S100A8 (pg/mL) | 15391.9(9692.3-20308.1) | 534.6(181.1-11275.1) | **＜0.001** |
| Haptoglobin (μg/mL) | 7.9(6.7-9.8) | 3.0(0.91-10.2) | **0.001** |
| Transthyretin (μg/mL) | 35.3(30.0-45.1) | 27.1(20.9-33.1) | **0.001** |

^$^Data are median (interquartile range, IQR).

^#^TBM includes definite and probable cases.

^&^Non-TBM includes viral meningitis, bacterial meningitis and cryptococcal meningitis.

TBM, tuberculous meningitis, n = 40; Non-TBM, n = 40, includes viral meningitis, bacterial meningitis and cryptococcal meningitis.
