# Supplementary figures and images for: Identification of protein biomarkers in host cerebrospinal fluid for differential diagnosis of tuberculous meningitis and other meningitis
Source: Front Neurol. 2022 Aug 8;13:886040. doi: 10.3389/fneur.2022.886040 (PMC9393334; doi:10.3389/fneur.2022.886040)

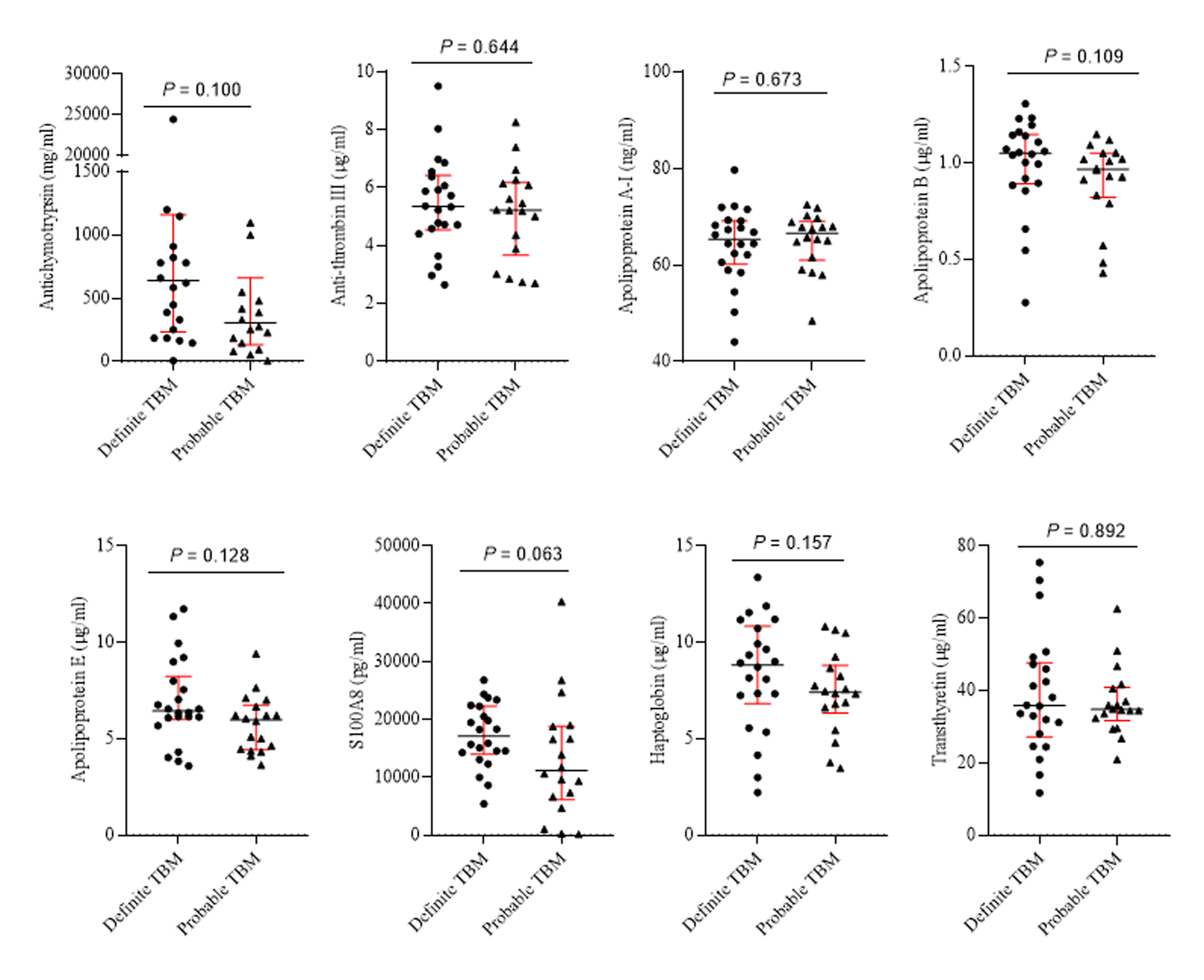

Supplement: Supplementary Figure 1 — The expression of 8 proteins in CSF between definite and probable TBM patients. [file Image_1.TIF]

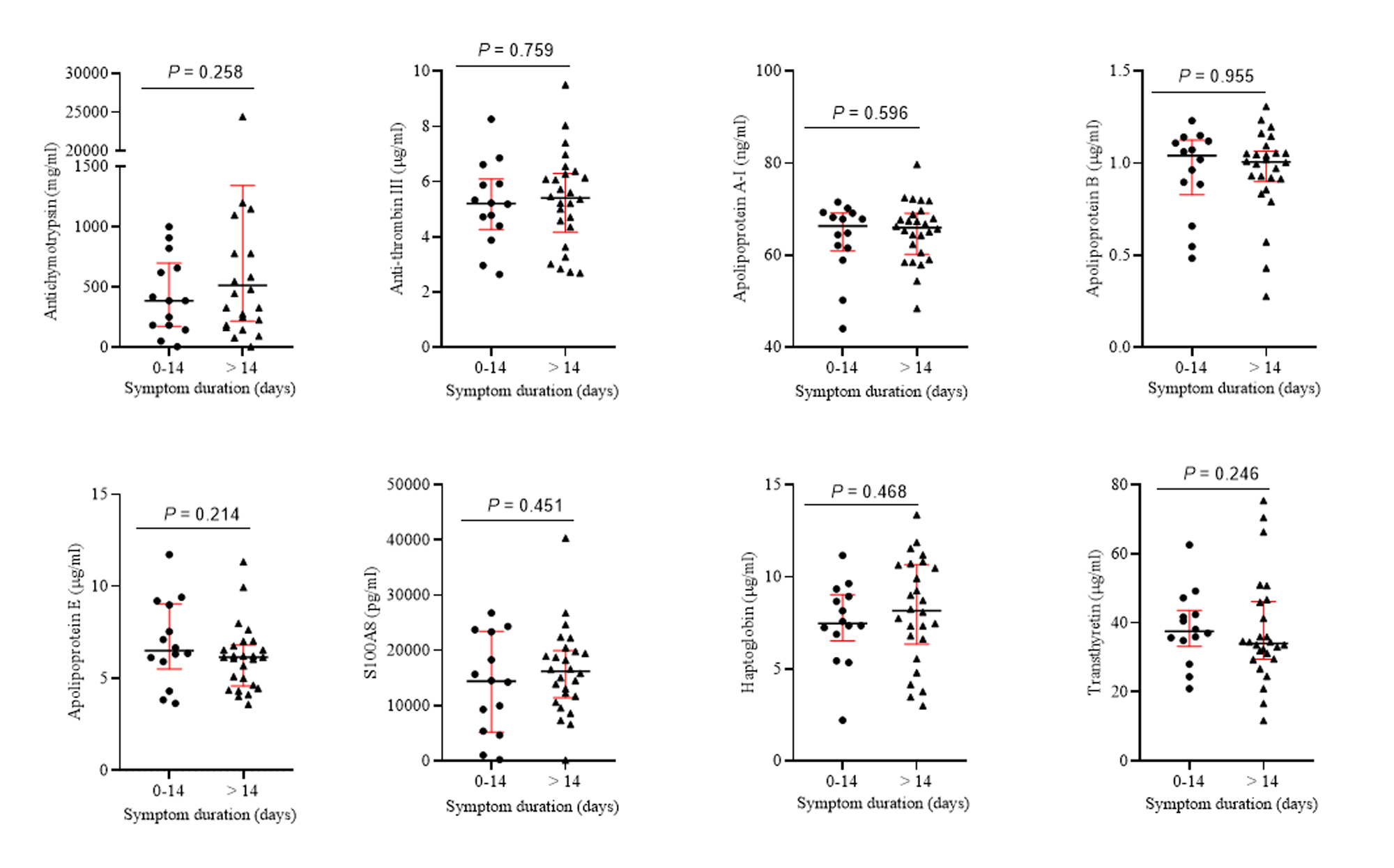

Supplement: Supplementary Figure 2 — The expression of 8 proteins in patients with TBM with different symptom durations. [file Image_2.TIF]

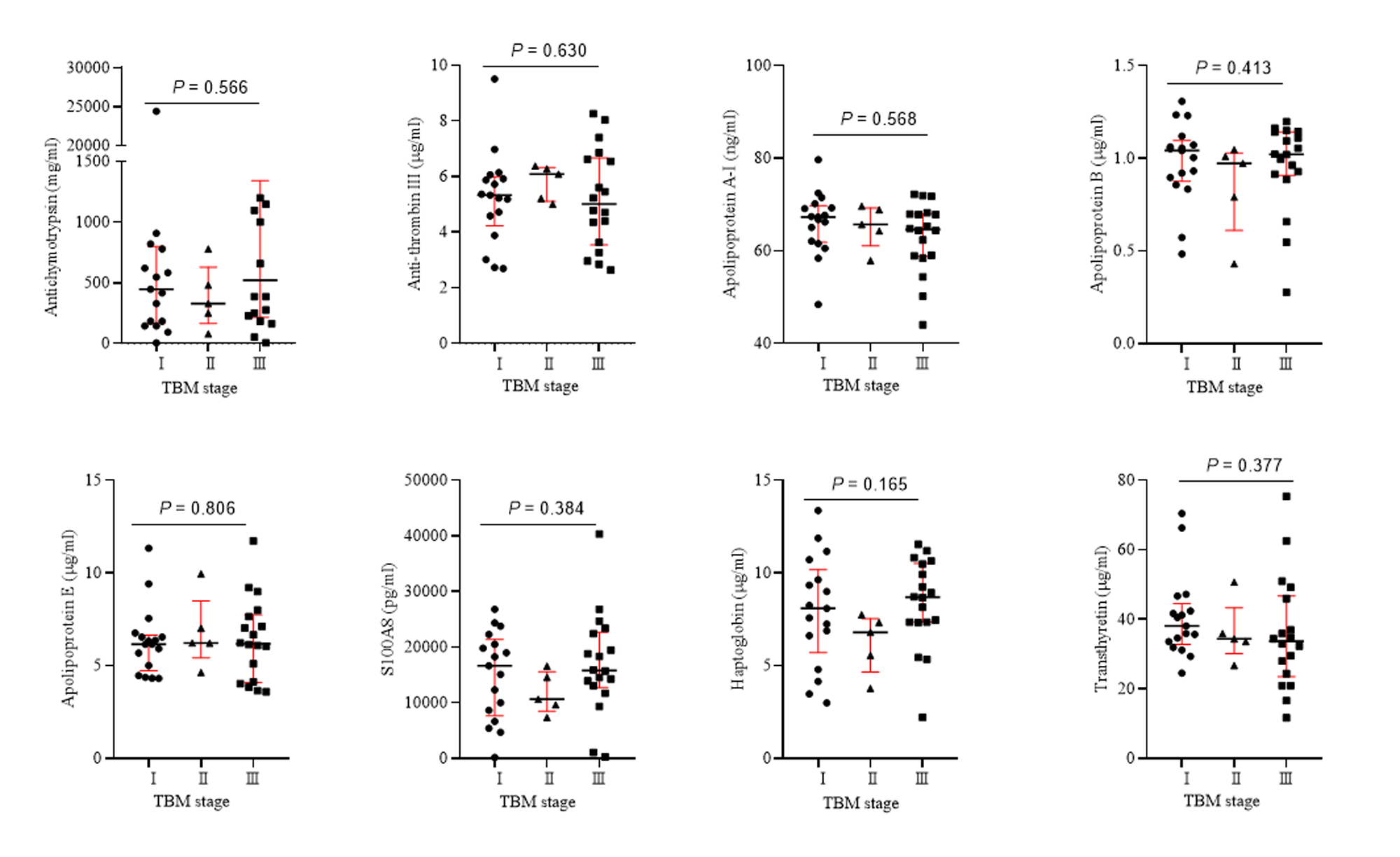

Supplement: Supplementary Figure 3 — The expression of 8 proteins in patients with TBM with different disease stages. [file Image_3.TIF]

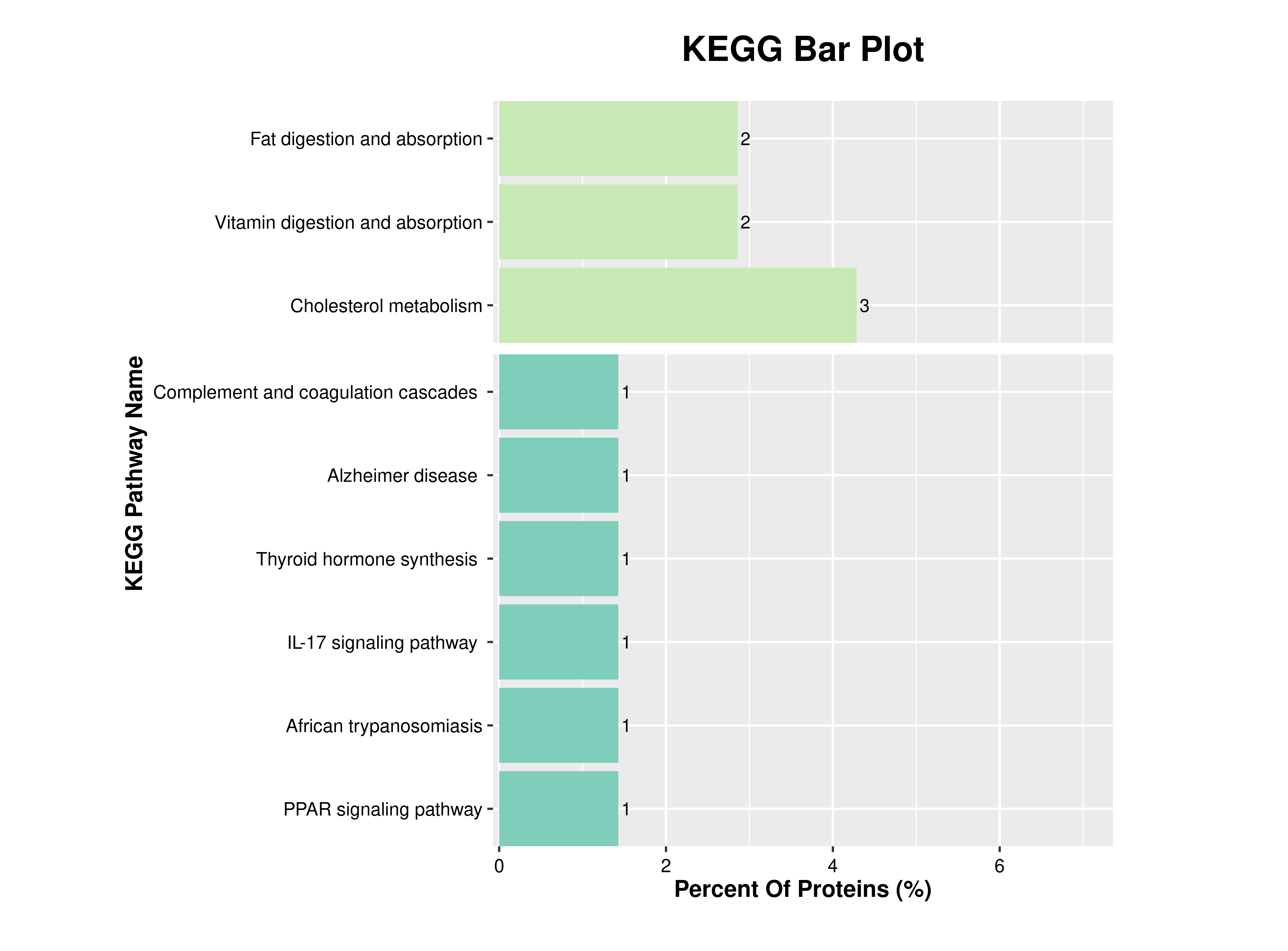

Supplement: Supplementary Figure 4 — KEGG analysis of 7 differentially expressed proteins. [file Image_4.TIFF]
